# Alcohol screening, brief intervention, and stepped care with older alcohol users

**DOI:** 10.1186/1940-0640-7-S1-A27

**Published:** 2012-10-09

**Authors:** Ruth McGovern, Simon Coulton, Jude Watson, Martin Bland, Colin Drummond, Eileen Kaner, Christine Godfrey, Alan Hassey, Dorothy Newbury-Birch

**Affiliations:** 1 Institute of Health and Society, Newcastle University, Newcastle upon Tyne, UK; 2 Center for Health Service Studies, University of Kent, Canterbury, UK; 3 Department of Health Sciences, University of York, Heslington, York, UK; 4 National Addiction Center, Institute of Psychiatry, King's College London, London, UK; 5 Fisher Medical Center, Skipton, UK

## 

The Alcohol Needs Assessment Project estimated that 20% of people aged ≥55 years consume alcohol at levels hazardous to their health, which is associated with a wide range of physical, psychological, and social problems, including coronary heart disease, hypertension, stroke, liver disease, and increased risk of a range of cancers. The Alcohol—Evaluating Stepped Care for Older Populations (AESOPS) research study is a randomized controlled trial looking at the effectiveness and cost-effectiveness of an opportunistic screening, brief intervention, and stepped care framework for older hazardous alcohol users in primary care compared with minimal intervention. Opportunistic screening of patients aged ≥55 years was conducted in 53 primary health care practices from eight areas across England. Patients who screened positive for an alcohol use disorder (AUD) were randomly allocated to one of two intervention conditions: brief structured advice (minimal intervention) or stepped care. Approximately 78,260 screening questionnaires were distributed, and 21,524 (27.5%) were returned. Seven-and-a-half percent of respondents screened positive for AUD. Of eligible patients, 51.3% were randomized to stepped care, most (99.6%) of whom received step one (brief lifestyle intervention); 55.1% received step two (brief motivational intervention), and 10.2% were referred to step three (specialist alcohol treatment). Results to date are discussed.

